# Validation of the Liverpool Elbow Score for evaluation of elbow stiffness

**DOI:** 10.1186/s12891-018-2226-0

**Published:** 2018-08-21

**Authors:** Ziyang Sun, Cunyi Fan

**Affiliations:** 1 Department of Orthopaedics, Shanghai Jiao Tong University Affiliated Sixth People’s Hospital, 600 Yishan Road, Shanghai, 200233 People’s Republic of China; 2 Department of Orthopaedics, Shanghai Sixth People’s Hospital East Affiliated to Shanghai University of Medicine & Health Sciences, Shanghai, People’s Republic of China

**Keywords:** Liverpool elbow score, Elbow stiffness, Scoring systems, Validation, Validity, Responsiveness

## Abstract

**Background:**

The Liverpool Elbow Score (LES) has been widely used to assess the outcomes of total elbow replacement in various conditions. However, there have been no published validation studies on LES for patients with stiff elbows undergoing arthrolysis. The purpose of this study was to find out whether LES could be equally applied to evaluate joint function in patients with elbow stiffness.

**Methods:**

A total of 63 patients with elbow stiffness were included in this retrospective validation study. The LES combines a nine-item patient-answered questionnaire (PAQ) and a six-item clinical assessment score (CAS), and can also be divided to evaluate two different parameters: elbow motion capacity (EMC) and elbow-related symptoms (ERS). Construct validity was assessed by correlating LES with previously validated scoring systems, and Spearman correlation coefficients (SCCs) were calculated. Effect size (ES) and standardized response mean (SRM) were calculated to determine responsiveness.

**Results:**

There were no ceiling or floor effects in the target population. Good-to-excellent validity was determined based on total score (0.45–0.89), PAQ (0.42–0.88), CAS (0.35–0.60), EMC (0.46–0.86), and ERS (0.36–0.59). High responsiveness (ES/SRM) was observed in total score (2.80/2.24), PAQ (2.34/1.78), CAS (2.90/2.34), EMC (2.92/2.35), and ERS (0.55/0.52).

**Conclusion:**

Our results suggest that the LES is a valid elbow-specific scoring system that can be used to evaluate joint function in patients with elbow stiffness, though some items included had some weakness either.

## Background

Elbow stiffness is a well-recognized disabling condition that causes functional impairment in the upper limb and interferes with daily activities. It is a very common complication after injuries or secondary to arthropathy, as both bony and soft tissue factors are the most important aetiologies [1–3]. Patients with limited elbow motion usually complain of difficulties in work, leisure activities, and even basic activities of daily living. Sometimes they are troubled with symptoms like pain, numbness, weakness, and instability. Clinical scoring systems are the most popular functional measurements used in the evaluation of orthopaedic patients. These systems are used to estimate the severity of dysfunction, evaluate treatment effectiveness, and compare different treatment methods [4, 5].

**Fig. 1:**
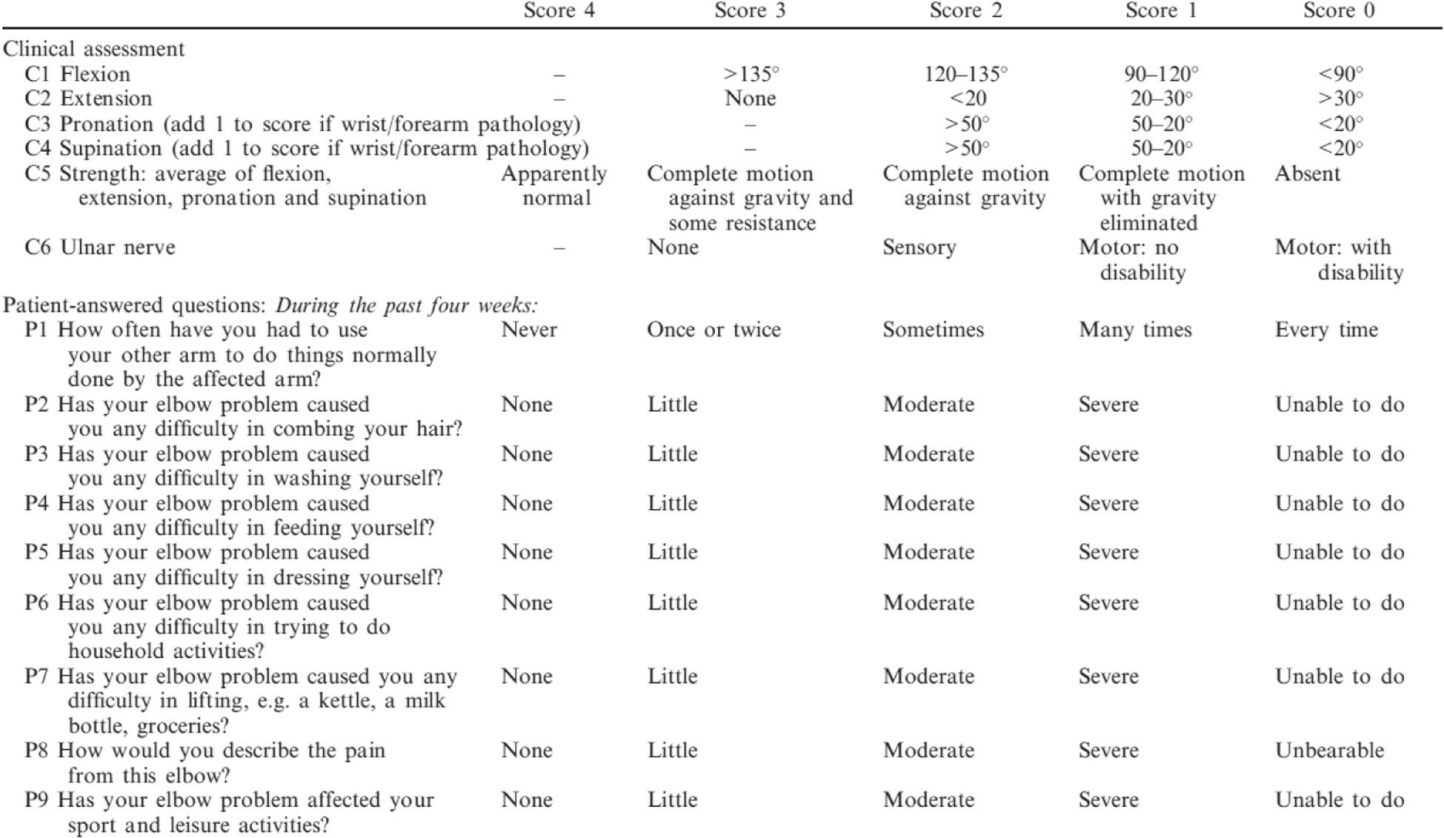
The components of the Liverpool Elbow Score

The Liverpool Elbow Score (LES, Fig. 1) was first introduced in 2004 as an elbow-specific outcome score to be completed by both the clinicians and patients. The LES combines a nine-item patient-answered questionnaire (PAQ, P1-P9) and a six-item clinical assessment score (CAS, C1-C6) [6]. The CAS comprises items that evaluate range of motion (C1-C4), muscle strength (C5), and ulnar nerve function (C6), whereas the PAQ assesses function and the ability to perform activities of daily living (P1-P7), levels of pain (P8), and participation in sporting and recreational activities (P9). The components of the LES, similar to most other elbow-related scoring systems, could also be divided into 2 parts comprising elbow motion capacities (EMC, C1-C4 and P1-P7 and P9) and elbow-related symptoms (ERS, C5-C6 and P8). ERS covers the items pertaining to muscle strength (C5) and ulnar nerve function (C6) from the CAS and pain (P8) from the PAQ. The remaining items form the EMC (C1-C4, P1-P7 and P9). In the original study, all items were measured on a scale from 0 to 10 and transformed in the calculation of the final score. The final scores were calculated as “final scores (LES) = (2/9) * (C1 + C2 + C3 + C4 + C6) + (1/6) * (C5 + P1 + P2 + P3 + P4 + P5 + P6 + P7 + P8 + P9)”, with values ranging from 0 to 10. The lower scores represented greater symptom and functional severity. Detailed item distributions in each of the different parts along with the individual score calculations and score ranges are shown in Table 1.

**Table 1:** Items distribution, scores calculation and score ranges of LES and different parts

Part	Items distribution	Scores calculation	Score ranges	
			Best	Worst
LES (total)	C1-C6, P1-P9	(2/9)* (C1 + C2 + C3 + C4 + C6) + (1/6)* (C5 + P1 + P2 + P3 + P4 + P5 + P6 + P7 + P8 + P9)	10	0
PAQ	P1-P9	(1/6)* (P1 + P2 + P3 + P4 + P5 + P6 + P7 + P8 + P9)	6	0
CAS	C1-C6	(2/9)* (C1 + C2 + C3 + C4 + C6) + (1/6)* (C5)	4	0
EMC	C1-C4, P1-P7, P9	(2/9)* (C1 + C2 + C3 + C4) + (1/6)* (P1 + P2 + P3 + P4 + P5 + P6 + P7 + P9)	8	0
ERS	C5-C6, P8	(2/9)* (C6) + (1/6)* (C5 + P8)	2	0

*LES* Liverpool Elbow Score, *PAQ* patient-answered questionnaire part, *CAS* clinical assessment score part, *EMC* elbow motion capacity, *ERS* elbow-related symptoms

After being demonstrated to be a reliable, valid and responsive outcome tool, the LES began to be used to assess outcomes after total elbow replacement in the management of rheumatoid arthritis [7, 8], posttraumatic arthritis [8–10], olecranon fractures [10] and distal humeral fractures [11, 12]. However, there have been no published validation studies of LES for patients with stiff elbows undergoing arthrolysis. Additionally, a well-established validation study might not be applicable to different populations, which means that a previously validated tool might have to be re-validated to justify its use in different populations [13]. Therefore, the purpose of this study was to determine whether the LES can be equally applied in different populations to evaluate joint function in patients with elbow stiffness.

## Methods

### Translation procedure

All the scoring systems (LES: Liverpool Elbow Score; DASH: Disability of arm, shoulder and hand questionnaire; OES: Oxford Elbow Score; MEPS: Mayo Elbow Performance Score; SF-36: Short Form-36) used in this study were translated into Simplified Chinese (Mainland) versions prior to having patients complete the questionnaires. Among these, MEPS has been widely used in China to evaluate elbow function, and validations of DASH and SF-36 have been performed in China [14, 15]. For LES and OES, however, there have been no other validation studies for these two scores in Simplified Chinese (Mainland) versions to this point.

Therefore, a 6-step method was used that included translation, synthesis, back-translation, expert committee review, pre-testing, and submission for appraisal, according to the guidelines of the cross-cultural adaptation process provided by Guillemin et al. [16, 17]. Briefly, the English versions of the LES and OES were translated separately by two native Chinese translators. A synthesized Simplified Chinese (Mainland) translation version was established after uniform agreement was reached between the two translators. The translated versions which was back-translated by two native English bilingual speakers who were blinded to the original English version. Then the four translators and two orthopaedic surgeons composed an expert committee that was established to compare the Chinese version to the original and back-translated versions. After an agreement on the semantic, idiomatic, experiential, and conceptual equivalence between the original and the target versions, and with an absence of language issues when the final version was pretested in 15 Chinese patients with elbow stiffness, the expert committee reached a consensus on the final version.

### Patients and study design

This was a retrospective validation study of patients who presented to our institution for elbow arthrolysis secondary to elbow stiffness between September 2016 and December 2016. Exclusion criteria were (1) unwillingness to participate or cooperate with follow-up; (2) illiteracy or an inability to comprehend the contents of the questionnaires; and (3) mental illness. All the patients underwent open arthrolysis by the same surgeon (C. F.) [18]. During the study period, 81 patients underwent surgery for elbow stiffness at our institution. Of these, 68 met the inclusion criteria. However, 5 of the 68 were excluded because of refusal or loss to follow up. The remaining 63 included patients were 45 men and 18 women, with a mean age of 35 years and a mean follow-up time of 13 months (other demographics and characteristics are shown in Table 2). The sample size of the respondents for validation of a scoring system was assumed to exceed three times the number of items in the system [19]. Therefore, with a total of 15 items, a total sample size of 63 was considered sufficient. All patients were asked to complete the patient-rated parts of LES, DASH, OES and SF-36. The physician-rated parts of LES and MEPS were assessed following a written protocol so that all the patients were examined using the same method.

**Table 2:** Demographics and clinical characteristics of patients

Characteristics	
No. of patients	63
Male	45 (71)
Age, years	35 ± 13 (11–62)
Height, cm	169 ± 9, (143–188)
Weight, Kg	66 ± 13, (32–105)
BMI, kg/m2	23.1 ± 3.4 (15.7–32.2)
Dominant arm	34 (54)
Follow-up time^a^, months	13 ± 1 (12–15)

Categorical variables are presented as number (%)

Continuous variables presented as mean ± standard deviation, (range)

*BMI* body mass index

^a^follow-up time means month post-operation from elbow release

### Testing and evaluation of measurement qualities

Floor and ceiling effects, reliability, construct validity, and responsiveness were required for a full validation of the scoring system [20, 21].

#### Reliability

Reliability measures whether the scores of the same patient show differences when implemented at different times or by different doctors (test-retest reliability), and whether the items in a domain have measured the same concept (internal consistency). However, this could not be measured due to the retrospective nature of our study.

#### Construct validity

Construct validity is defined as the degree to which the scores of a particular instrument are related to a gold standard test. Unfortunately, no gold standard test has been established to reflect pre- and post-arthrolysis status. The DASH can be used to measure disability in any region of the upper limb and has been shown to be valid and responsive compared to other joint-specific measures of the upper extremity, and comprises 2 parts (Disability and Symptoms) [22]. The OES was reported to be a valid, reliable, and responsive self-administered instrument that can be used for several types of elbow function measurements, and it comprise 3 parts (Pain, Elbow Function and Social-psychological) [23]. The DASH and OES have been shown to correlate to general health measures such as the SF-36 [23, 24]. Consisting of physician-rated pain, ROM, stability, and a patient-rated daily function, MEPS [25] was the most widely used elbow function assessment, according to a systematic review including 980 studies and exploring trends and distributions of clinical rating systems in elbow research [26]. Construct validity was assessed by correlating LES to DASH, OES, MEPS, and SF-36 (PCS and MCS) in total scores (TOTAL), PAQ, CAS, EMC and ERS. Spearman’s correlation coefficients (SCCs) were calculated. In this study, the Disability portion of DASH, the Elbow Function portion of OES, and the range of motion and daily activity function portion of MEPS comprised the EMC. The symptom portion of DASH, the pain portion of OES, and the pain and stability portions of MEPS comprised the ERS. The TOTAL, PAQ, and CAS portions of the LES were correlated with the TOTAL portions of DASH, OES, MEPS, and SF-36 (PCS and MCS). EMC and ERS portions of the LES were correlated with EMC and the ERS portions of DASH, OES and MEPS, as well as the TOTAL part of SF-36 (PCS and MCS).

#### Responsiveness

Responsiveness measures the sensitivity in changes in preoperative and follow-up results. In our study, the effect size (ES) and standardized response mean (SRM) were calculated for the TOTAL part of LES, DASH, OES, MEPS, and SF-36 (PCS and MCS) and the EMC and ERS parts of LES, DASH, OES, MEPS, as well as CAS and PAQ parts of LES. The ES was calculated as the mean difference between the baseline scores and the follow-up scores divided by the standard deviation of the baseline scores [27]. The SRM was calculated as the mean change in the scores divided by the standard deviation of the change in scores [28].

### Statistical analyses

All statistical analyses were performed using IBM SPSS, Version 22.0 (IBM Corp, Armonk, NY, USA). Categorical data are presented as numbers (percentages). Continuous data are presented as means ± standard deviations (range). *P* values of less than 0.05 were considered statistically significant. Floor or ceiling effects existed when more than 15% of the patient collective achieved the highest or lowest possible score on the LES [29].SCC was considered strong for construct validity if the value was greater than 0.5, moderate if the value was between 0.5 and 0.35, and weak if the value was less than 0.35 [30]. An ES of 0.2 to 0.5 reflected small responsiveness, 0.5 to 0.8, moderate responsiveness, and greater than 0.8, large responsiveness, as well as SRM [31].

## Results

All patients completed the PAQ with no difficulties and with no items missing or showing multiple responses. No floor or ceiling effects were found in the target population (Table 3). All of the SCCs were positive except the relationship with DASH, which was scored in a different direction (Table 4). The LES overall scores correlated well with all the compared total scores (*p* < 0.001 for all), as DASH (*r* = 0.89 preoperatively and 0.86 post-operatively), OES (*r* = 0.83 and 0.79), MEPS (*r* = 0.66 and 0.49), SF-36 (PCS, *r* = 0.65 and 0.64; MCS, *r* = 0.45 and 0.68), as well as with the PAQ and CAS parts of LES. The EMC and ERS parts of LES correlated either strongly or moderately with similar parts of DASH, OES, and MEPS as well as with SF-36/PCS and SF-36/MCS preoperatively and postoperatively. All in all, the different LES parts also correlated well with DASH, OES, MEPS, and SF-36, with either high or moderate correlations in TOTAL (0.45–0.89), PAQ (0.42–0.88), CAS (0.35–0.60), EMC (0.46–0.86), and ERS (0.36–0.59).

**Table 3:** Floor and ceiling effects of LES

Component	LES (No.^a^)		Floor effect (%^b^)	Ceiling effect (%^b^)
	Lower limit	Upper limit		
TOTAL	2.3 (1)	9.1 (1)	0	0
PAQ	0.8 (1)	6.0 (1)	0	0
CAS	1.1 (1)	3.1 (2)	0	1.6
EMC	0.8 (1)	7.1 (1)	0	0
ERS	1.1 (1)	2.0 (4)	0	6.4

*LES* Liverpool Elbow Score, *TOTAL* total scores, *PAQ* patient-answered questionnaire part, *CAS* clinical assessment score part, *EMC* elbow motion capacity, *ERS* elbow-related symptoms

^a^Number of patients showing the lowest or highest values in various parts;

^b^Percent of patients achieving the lowest or highest values in various parts

**Table 4:** Construct validity. Spearman Correlation Coefficients (SCCs) between LES and DASH, OES, MEPS and SF-36

	TOTAL	PAQ	CAS	EMC	ERS
Preoperative data					
DASH	0.89^***^	0.88^***^	0.44^***^	0.86^***^	0.54^***^
OES	0.83^***^	0.82^***^	0.45^***^	0.82^***^	0.51^***^
MEPS	0.66^***^	0.65^***^	0.38^**^	0.67^***^	0.36^**^
SF-36/PCS	0.65^***^	0.63^***^	0.42^**^	0.60^***^	0.45^***^
SF-36/MCS	0.45^***^	0.42^**^	0.35^**^	0.50^***^	0.41^**^
Follow-up data					
DASH	0.86^***^	0.87^***^	0.57^***^	0.72^***^	0.59^***^
OES	0.79^***^	0.87^***^	0.46^***^	0.69^***^	0.54^***^
MEPS	0.49^***^	0.53^***^	0.35^**^	0.46^***^	0.43^***^
SF-36/PCS	0.64^***^	0.50^***^	0.52^***^	0.52^***^	0.50^***^
SF-36/MCS	0.68^***^	0.50^***^	0.60^***^	0.60^***^	0.50^***^

*LES* Liverpool Elbow Score, *TOTAL* total scores, *PAQ* patient-answered questionnaire, *CAS* clinical assessment score, *EMC* elbow motion capacity, *ERS* elbow-related symptoms, *DASH* Disability of arm, shoulder and hand questionnaire, *OES* Oxford Elbow Score, *MEPS* Mayo Elbow Performance Score, *SF-36/PCS* physical component summary part of Short Form-36, *SF-36/MCS* mental component summary part of Short Form-36, *SCCs* Spearman Correlation Coefficients

**P < 0.01, ***P < 0.001

The LES was found to be more responsive (change from preoperative to follow up) than all the compared scores: DASH, OES, MEPS and SF-36 scores (Table 5). LES showed a large (ES/SRM > 0.8/0.8) responsiveness for TOTAL (2.80/2.24, *p* < .001), and all parts of the PAQ, CAS, and EMC (except for ERS with a moderate responsiveness of 0.55/0.52, *p* = .001). This analysis also showed that LES was more responsive than DASH with an ES/SRM of 1.96/1.51 (*p* < .001), OES of 2.12/1.65 (*p* < .001), MEPS of 2.32/1.72 (*p* < .001) and SF-36 (PCS, 1.28/0.83, *p* < .001 and MCS, 1.28/0.98, *p* < .001).

**Table 5:** Responsiveness of LES compared with DASH, OES, MEPS and SF-36

Questionnaires	Mean (SD)			*P* value	ES	SRM
	Preoperative	Follow-up	Change			
LES						
TOTAL	5.7 (1.3)	8.8 (0.8)	3.1 (1.4)	< 0.001	2.80 (L)	2.24 (L)
PAQ	3.4 (1.1)	5.5 (0.5)	2.1 (1.2)	< 0.001	2.34 (L)	1.78 (L)
CAS	2.3 (0.4)	3.3 (0.3)	1.0 (0.4)	< 0.001	2.90 (L)	2.34 (L)
EMC	3.9 (1.2)	6.9 (0.7)	3.0 (1.3)	< 0.001	2.92 (L)	2.35 (L)
ERS	1.8 (0.2)	1.9 (0.2)	0.1 (0.2)	0.001	0.55 (M)	0.52 (M)
DASH						
TOTAL	35 (18)	8 (9)	27 (18)	< 0.001	1.96 (L)	1.51 (L)
EMC	27 (15)	5 (8)	22 (15)	< 0.001	1.90 (L)	1.48 (L)
ERS	7 (4)	3 (2)	4 (4)	< 0.001	1.41 (L)	1.04 (L)
OES						
TOTAL	61 (16)	88 (9)	27 (17)	< 0.001	2.12 (L)	1.65 (L)
EMC	65 (19)	95 (11)	30 (19)	< 0.001	1.99 (L)	1.61 (L)
ERS	74 (19)	86 (11)	13 (20)	< 0.001	0.81 (L)	0.63 (M)
MEPS						
TOTAL	65 (12)	88 (7)	23 (14)	< 0.001	2.32 (L)	1.72 (L)
EMC	23 (10)	44 (2)	22 (10)	< 0.001	2.87 (L)	2.11 (L)
ERS	42 (8)	44 (6)	2 (8)	0.064	0.26 (S)	0.21 (S)
SF-36						
PCS	60 (17)	82 (17)	22 (26)	< 0.001	1.28 (L)	0.83 (L)
MCS	53 (20)	78 (18)	24 (25)	< 0.001	1.28 (L)	0.98 (L)

*LES* Liverpool Elbow Score, *TOTAL* total scores, *PAQ* patient-answered questionnaire, *CAS* clinical assessment score, *EMC* elbow motion capacity, *ERS* elbow-related symptoms, *DASH* Disability of arm, shoulder and hand questionnaire, *OES* Oxford Elbow Score, *MEPS* Mayo Elbow Performance Score, *PCS* physical component summary part, *MCS* mental component summary part, *SD* standard deviation, *ES* effect size, *SRM* standardized response mean

(L) a large responsiveness, ES of greater than 0.8; (M) a moderate responsiveness, ES of 0.5 to 0.8; and (S) a small responsiveness, ES of less than 0.5; as well as SRM

## Discussion

The most important finding of this study was that the LES was a valid elbow-specific scoring system to evaluate joint functions in patients with elbow stiffness, and contains both subjective and objective parameters. It is based on a 15-item tool with a scale ranging from 0 to 10 points, with higher scores indicating better function.

The LES was simple enough to be rapidly administered in clinics and there were no ceiling or floor effects in our study, which demonstrated that the distribution of LES was satisfactory. Regrettably, reliability could not be measured due to the retrospective study design. Construct validity and responsiveness were assessed for validation. Because no gold standard measurement had been established for comparison of the construct validity between elbow scores, correlations (SCCs) of LES with previously validated scoring systems were determined by 0.44–0.89 for DASH and 0.35–0.67 for MEPS. In fact, validity was shown by good correlations with DASH (*r* = 0.79; *r* = 0.89 preoperatively and 0.86 postoperatively in our study) and NHP (Nottingham Health Profile, *r* = 0.54) in the original publication study for arthritis [6]. Additionally, a good correlation (SCC, 0.84; 0.66 in this study) was also shown with MEPS for patients undergoing total elbow arthroplasty [8]. Currently, the method of choice to determine responsiveness remains unknown, though various statistics are available [32]. The determination of the effective size (ES) and standardized response mean (SRM) in addition to the Global Perceived Effect (GPE) Score was considered to be an appropriate improvement to assess responsiveness [33]. Due to the retrospective nature of the study, ES and SRM were calculated and a large responsiveness was found in LES, which were larger than DASH, OES, MEPS, and SF-36 in our study. The responsiveness of LES was found to correlate well with DASH (*r* = 0.45; 0.85 in our study) and NHP (*r* = 0.42) in the original study [6]. LES was also found to have large ES (1.64; 2.80 in our study), SRM (1.25; 2.24 in our study) and GRR (Guyatt responsiveness ratio, 1.69) during the follow-up period for patients undergoing elbow arthroplasty [32]. Interestingly, we found a lower responsiveness in ERS compared to DASH and OES. Our explanation for this difference was that there were extra stiffness items and quality of life items in the ERS of DASH, and the ERS of OES contains only items for pain, which would contribute to the bias of the comparison.

Recently, self-assessment scores in outcome studies are becoming more and more popular due to their financial and logistic advantages [34]. However, leaving objective parameters out might miss important aspects of elbow pathology that are important in symptom assessment and are impossible to evaluate by only using a self-assessment score. These aspects include elbow instability, reduced muscle strength, and nerve dysfunction. In fact, functions and symptoms in an individual joint may not be evaluated accurately by subjective questionnaires alone [35]. The questions presented to patients are also sometimes lengthy and not relevant to specific problems [36]. Objective parameters alone have been also found to have no correlation with patient’s satisfaction [37], life background, since expectations and satisfaction are different for different individuals. Therefore, it is preferable for the LES to be used to evaluate the joint functions of patients with elbow stiffness by using self-assessment questionnaires in addition to physician-assessment parameters.

However, there are also some weaknesses that need to be realized when using the LES to evaluate joint function in patients with elbow stiffness. The researchers that invented the LES decided to remove the instability test from the objective parameters as they thought it was associated with a rare elbow problem. When presented, it would have such a massive impact on elbow function that it would be easily detected [6]. However, according to our clinical experience, we believe that elbow instability is a perfect sign of collateral ligament dysfunction, which is a common complication in elbow trauma, and an indication for surgical therapeutic options and postoperative rehabilitation. Therefore, it would be better if instability was considered. For measuring strength as an elbow specific function, the MRC scale was used in most systems, as was the LES, which is a subjective qualitative assessment made by the surgeons. However, L Shahgholi. found that half of the patients clinically assessed as having normal (5/5) elbow flexion strength on manual muscle testing exhibited less than 42% of their age-expected strength on quantitative testing, as well as elbow extension strength testing. They concluded that even when performed by experienced clinicians, manual muscle testing may be more misleading than expected for subjects graded as having normal (5/5) strength [38, 39]. Therefore, measuring strength with a dynamometer would be a more objective and responsive measure than measuring strength with the MRC scale, and it could be measured over time and compared to normative data. Strength associated with grip, elbow and wrist motion are all necessary in assessing elbow function, especially in patients planning for elbow arthrolysis surgery, as reduced muscle strength is a common complication after arthrolysis [40]. Additionally, pain has a strong impact on elbow function and health status measures [41]. Due to the strong influence of psychological and sociological factors on the experience of pain, the expression of pain should probably be evaluated separately from objective parameters in physician-rated domains [42]. Though the expression of pain is obtained from the PAQ portion of the LES, it comprises only 1/9 (~ 11%) of the PAQ and 1/15 (~ 7%) of the whole scores, which is in contrast to most of other scoring systems, in which pain is weighted as 30–50% of the total score [4, 5]. In fact, a five-level Likert scale could not fully generalize the expression of pain from patients and detect its changes from pre- to post-operation. We believe that these limitations may also contribute to its moderate responsiveness in the ERS. Finally, according to the International Classification of Functioning, Disability and Health (ICF), health and disability would be better measured in three domains: physician-rated body functions and structures, patient-rated activities and participation, and patient-rated quality of life [43]. Unfortunately, LES does not include items inquiring about patients’ qualities of life.

This study has some weaknesses. The biggest limitation of this study was that the test-retest reliability and internal consistency could not be validated due to the retrospective nature of the study, which is an important step (i.e. reliability) in evaluating a scoring system. The retrospective study could contribute to the bias in the validated results. Another limitation is that as it was a single-centre study, and it could not be said with certainty that these results could be applied to other centres. Therefore, further prospective research with a larger population from multiple clinical centres is needed.

## Conclusion

Based on the present data, our results suggested that the LES is a valid elbow-specific scoring system and is applicable to evaluate joint functions of patients with elbow stiffness, although some items included had some weakness either. Further prospective research using a larger population from multiple clinical centres is required in future.

## Abbreviations

CAS: Clinical assessment score
DASH: Disability of arm, shoulder and hand questionnaire
EMC: Elbow motion capacity
ERS: Elbow-related symptoms
ES: Effect size
LES: Liverpool Elbow Score
MEPS: Mayo Elbow Performance Score
OES: Oxford Elbow Score
PAQ: Patient-answered questionnaire
SCC: Spearman correlation coefficients
SF-36: Short Form-36
SF-36/MCS: Mental component summary part of Short Form-36
SF-36/PCS: Physical component summary part of Short Form-36
SRM: Standardized response mean
